# Clinical Experience with the M4 Ahmed Glaucoma Drainage Implant

**DOI:** 10.5005/jp-journals-10028-1231

**Published:** 2017-10-27

**Authors:** Ilya Sluch, Brett Gudgel, Justin Dvorak, Mary Anne Ahluwalia, Kai Ding, Steve Vold, Steven Sarkisian

**Affiliations:** 1 Ophthalmologist, Department of Ophthalmology, Dean McGee Eye Institute Oklahoma City, Oklahoma, USA; 2 Ophthalmologist, Department of Ophthalmology, Dean McGee Eye Institute Oklahoma City, Oklahoma, USA; 3 Biostatistician, Department of Biostatistics, University of Oklahoma, Oklahoma City, Oklahoma, USA; 4 Ophthalmologist, Department of Ophthalmology, Triad Eye Institute, Tulsa Oklahoma, USA; 5 Biostatistician, Department of Biostatistics, University of Oklahoma, Oklahoma City, Oklahoma, USA; 6 Ophthalmologist, Department of Ophthalmology, Vold Vision, Fayetteville Arkansas, USA; 7 Ophthalmologist, Department of Ophthalmology, Dean McGee Eye Institute Oklahoma City, Oklahoma, USA

**Keywords:** Ahmed valve, Drainage device, M4.

## Abstract

**Aim:**

To evaluate the safety and efficacy of the M4 (porous polyethylene plate) Ahmed Glaucoma Valve (AGV) drainage implant in a multicenter retrospective study.

**Materials and methods:**

A retrospective chart review of medical records of patients who had undergone the M4 Ahmed valve was performed from January 2013 to April 2015. The primary outcome measure was surgical failure defined as: Less than a 20% reduction in baseline intraocular pressure (IOP) to last follow-up visit, final IOP less than 5 mm Hg or greater than 18 mm Hg, reoperation for glaucoma, or loss of light perception vision. All eyes not meeting the above criteria were defined as success.

**Results:**

A total of 291 eyes met all study inclusion criteria. The average follow-up in the study was 6 months (±7.6 months) with 112 patients achieving 12-month follow-up (38.5%). 208 eyes (71.5%) met the study success criteria at final follow-up. No statistically significant spikes in postoperative IOP at 1 and 4 months were detected. The average preoperative IOP was 26.0 on an average of 2.8 medications. At 6 months, the average IOP dropped to 16.7 on 0.9 medications and stayed relatively stable at 15.8 on 1.2 medications at 12-month follow-up.

**Conclusion:**

The M4 valve appears to have less of a hypertensive phase compared with the other Ahmed class valves with a similar safety profile. While 71.5% success rate was achieved at final follow-up, the failure rate steadily increased over time.

**Clinical significance:**

While the M4 production has been discontinued, the porous design of the M4 may avoid a pressure spike in the Ahmed valve class and warrants future investigation for valve design.

**How to cite this article:** Sluch I, Gudgel B, Dvorak J, Ahluwalia MA, Ding K, Vold S, Sarkisian S. Clinical Experience with the M4 Ahmed Glaucoma Drainage Implant. J Curr Glaucoma Pract 2017;11(3):92-96.

## INTRODUCTION

The AGV (New World Medical Inc., Rancho Cucamonga, CA) is an intraocular implant used to lower the pressure in the eye by creating a shunt for the aqueous humor to flow to the extraocular surface. A small caliber tube is traditionally placed into the anterior chamber to allow the fluid to drain to the surface into a subconjunctival bleb fluid pocket created via the plastic plate of the device. Aqueous fluid is thought to drain into the bleb and get absorbed by the surrounding tissue. To prevent postoperative hypotony due to a sudden reduction of pressure, the Ahmed implant has a flow-resistance valve that maintains the IOP above 8 mm Hg.^1^

Excessive scarring of the bleb is thought to impede the flow and aqueous absorption leading to device failure. Histology studies showed that the filtering bleb created under the conjunctiva forms an inner layer of compressed collagen and an outer densely vascularized layer.^2^ Previous studies failed to show improved success using antimetabolite drugs to target fibroblast proliferation in glaucoma tube drainage devices.^3^ However, studies have shown improved function in devices that have a more vascular and flexible capsule, suggesting more benefit in a device with a thinner inner collagen layer and more vascularization.^4-6^

A new M4 design of the Ahmed valve utilizes a porous high-density polyethylene polymer whose pores allow for tissue integration and vascular ingrowth with animal models showing a thinner and more vascular capsule.^7-9^ A recent study comparing the M4 valve with the traditional S2 and FP7 showed no difference in final pressure outcomes.^10^ The M4 valve did not have a “hypertensive spike” phase several months after implantation but had similar IOPs compared with the other two valves at follow-up.^10^

We present our experience with the M4 valve.

## MATERIALS AND METHODS

A retrospective chart review was carried out on patients who had undergone glaucoma drainage implant surgery with the M4 Ahmed valve from January 2013 to April 2015 at the Dean McGee Eye Institute (DMEI), Oklahoma City, OK, USA, and Vold Vision, Fayetteville, AR, USA. Age, ethnicity, sex, glaucoma type, previous eye surgical history, pre- and postoperative IOPs, vision, number of glaucoma medications at follow-up, and complications were collected over the course of the study. This study was approved by the University of Oklahoma Institutional Review Board.

The primary outcome measure was surgical failure defined as less than a 20% reduction in baseline IOP to last follow-up visit, final IOP less than 5 mm Hg or greater than 18 mm Hg, reoperation for glaucoma, or loss of light perception vision. All eyes not meeting the above criteria were defined as success.

Secondary outcome measures included IOP at each follow-up visit, number of postoperative medications, and best-corrected visual acuity. The statistical significance of the median change from baseline for each follow-up period was assessed using the Wilcoxon signed-rank test.

The hypertensive spike phenomenon was defined as a transient elevation in IOP at the 1-month or 4-month visits. To assess for a postoperative spike in IOP, the difference between IOP at each time point and final IOP was computed for each patient, and then subjected to a series of paired t-tests.

For the Kaplan-Meier analysis, surgical failure was defined as a final IOP less than 5 mm Hg or greater than 21 mm Hg, and/or a <20% reduction in IOP from baseline, following Cvintal et al.^11^ Time to event was defined as the follow-up period. If a patient dropped out or the follow-up period ended before surgical failure occurred, his/her time-to-failure data were considered right-censored. Analysis via Kaplan-Meier curves was performed to estimate mean and median time to surgical failure. Survival denotes a patient continuing in the study without experiencing surgical failure.

## RESULTS

A total of 316 eyes of 284 patients were identified to have undergone the M4 Ahmed Valve implant from January 2013 to April 2015 at the DMEI and Vold Vision. A total of 25 eyes (17 DMEI and 8 Vold Vision) were excluded due to follow-up less than 1 month or insufficient preoperative medical records, with the remaining 291 eyes undergoing analysis. Preoperative demographics and characteristics are shown in Table 1.

The average follow-up in the study was 6 months (±7.6 months) with 112 patients achieving 12-month follow-up (38.5%), 71 achieving 18-month follow-up (24.4%), and 28 achieving 24-month follow-up (9.6%). A total of 208 eyes (71.5%) met the study success criteria (Table 2). No statistically significant spike in postoperative IOP was detected. However, IOPs immediately postoperative, 1 week post, and 1 month post were significantly lower than final IOP.

**Table 1:** **Table 1:** Patient demographics

*Age (years)*		*69.7 ± 15.9 (17.0-101)*			
*Follow-up time (months)*		*6.0 ± 7.6 (1-30)*			
		*n*		*Percent*	
Sex					
Male		143		49.1	
Female		148		50.9	
Race					
White		251		86.3	
African-American		23		7.9	
Asian		3		1.0	
Hispanic		14		4.8	
Diagnosis					
POAG		197		67.7	
NVG		26		8.9	
Uveitic		26		8.9	
UGH		4		1.4	
Traumatic		9		3.1	
Congenital		5		1.7	
CACG		10		3.4	
PSX		6		2.1	
Other OAG		6		2.1	
Pigmentary		3		1.0	
Steroid induced		1		0.3	
ICE syndrome		2		0.7	
NTG		2		0.7	
Previous tube surgery					
Yes		10		3.4	
No		281		96.6	
Previous trabeculectomy					
Yes		82		28.2	
No		209		71.8	
Other previous surgery					
Yes		239		82.1	
No		52		17.9	

Patient Demographics. POAG: Primary open angle glaucoma; NVG: Neovascular glaucoma; UGH: Uveitis-glaucoma hyphema syndrome; CACG: Chronic angle closure glaucoma; PSX: Pseudoexfoliation glaucoma; OAG: Open angle glaucoma; ICE Syndrome: Iridocorneal endothelial syndrome; NTG: Normal tension glaucoma

**Table 2:** **Table 2:** Surgical outcomes breakdown at final visit

21 ≥ IOP ≥ 5		225/291		77.3%	
18 ≥ IOP ≥ 5		199/291		68.4%	
>20% in IOP		199/291		68.4%	
Overall study success		208/291		71.5%	
Conversion to NLP		3/291		1.0%	

Overall study success defined as 18 ≥ IOP ≥ 5 or >20% reduction in IOP without conversion to “no light perception” (NLP) vision

**Graph 1:**
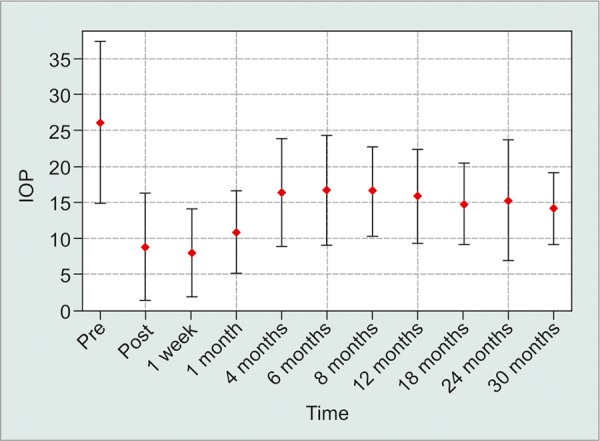
Average IOP within a standard deviation

**Graph 2:**
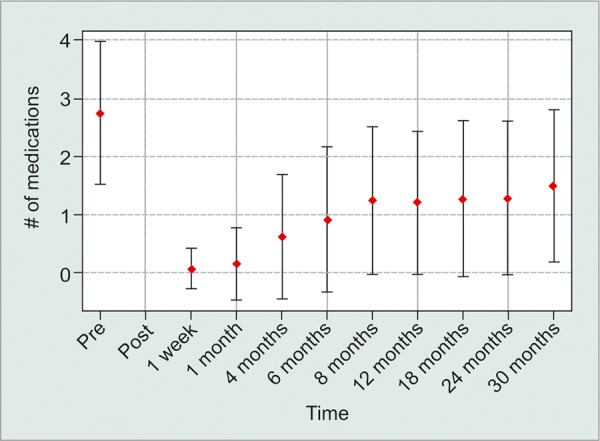
Average number of medications within a standard deviation

**Graph 3:**
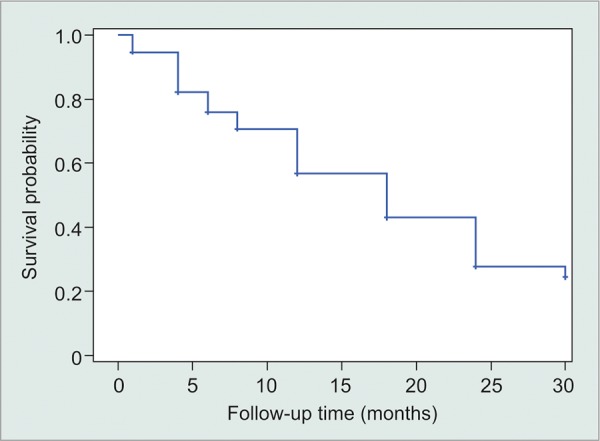
Survival denotes a patient continuing in the study without experiencing surgical failure

The average preoperative IOP was 26.0 on an average of 2.8 medications. At 6 months, the average IOP dropped to 16.7 on 0.9 medications and stayed relatively stable at 15.8 on 1.2 medications at 12-month follow-up. The average IOP appeared to be sustained below 16 throughout the study period after the first 12 months, with a modest increase in the number of medications (Graphs 1 and 2). The visual acuity appeared stable throughout the study. By Kaplan-Meier analysis, mean time to surgical failure was 17.4 ± 0.8 months; however, this was underestimated because the largest event time was censored (Graph 3).

**Graph 4:**
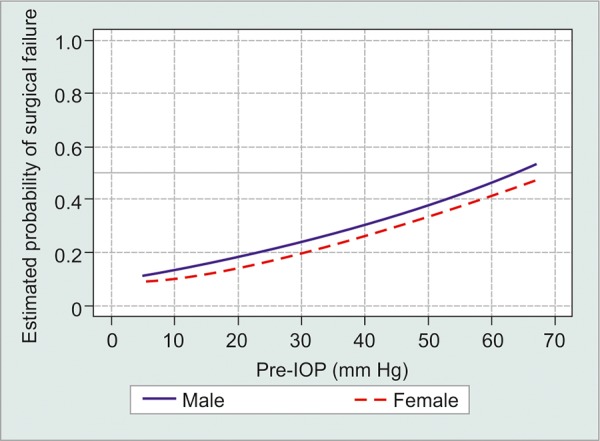
Probability of surgical failure as a function of preoperative IOP

Immediate postoperative complications included hypotony (69, 23.7%), hyphema (50, 17.2%), corneal edema (37, 12.7%), tube exposure (17, 5.8%), bleb leak (12, 4.1%), and tube occlusion (11, 3.8%). Chronic hypotony developed in 15 patients (5.2%). Complications were more commonly associated with increased age [odds ratio 1.02, confidence interval (CI) 1.0-1.04, p < 0.016] and elevated preoperative IOP (odds ratio 1.05, CI 1.03-1.08, p < 0.0001, Graph 4). A total of 30 patients required surgical intervention for tube exposure (17, 5.8%), tube occlusion (6, 2.1%), bleb leak (4, 1.4%), hyphema washout (2, 0.7%), choroi-dal drainage (1, 0.3%). A total of 41 patients required further intervention including cyclophotocoagulation endoscopic cyclophotocoagulation (CPC/EPC) (30, 10.3%), filtration surgery/tube (8, 2.7%), and enucleation (3, 1.0%). No statistically significant difference in complications or outcomes was seen among the two centers.

## DISCUSSION

The M4 valve was able to obtain a greater than 10 mm Hg drop in IOP postoperatively and decrease in the number of medications by more than half at the 12-month follow-up. This was sustained throughout the duration of the study with 71.5% of patients achieving surgical success at final follow-up. However, based on the Kaplan-Meier analysis, the rate of failure steadily increased over time. Our results are very similar to Kim et al’s^10^ study at 18 months (study duration) comparing all three Ahmed valves. This suggests that glaucoma drainage implants fail over time in a similar fashion. At our institution, glaucoma drainage implants are reserved for the most refractory glaucoma patients, which may contribute to the high failure rate.

On average, the M4 valve creates a significant drop in IOP postoperatively with a slow rise of around 16 mm Hg by the 4-month follow-up. The hypertensive phase was not detected in our study compared with other studies of the different Ahmed drainage implants.^10,12,13^ Based on our protocol, since the follow-up was scheduled at 1 week, 1 month, and 4 months, it is possible that the hypertensive phase was missed in-between visits. However, our data still suggest that the hypertensive phase is less with the M4 compared with the other Ahmed class valves, since the previously reported hypertensive phases in other trials were also reported in the first few months.^10-13^

At 1-year follow-up, our patients, on average, were able to achieve an IOP of 15.8, which is consistent with the literature range of IOP obtained with other Ahmed valves (FP7: 13.8-17.7, S2: 15.8-17.7, M4: 15.0-20.3).^10,11,14-17^ About 5.2% of patients developed chronic hypotony and 10.3% of patients required further surgical intervention for IOP lowering, most commonly CPC/ECP. The device appears to have an average complication rate in the Ahmed class requiring revision or device removal (hypotony: 3-13%, removal/revision: 0-15.4%).^10,14-17^

The IOP effect appears to be sustained through the 30-month follow-up in our study, with no detectable hypertensive phase at the 1-month or 4-month visits. Kim et al’s^10^ study defined a hypertensive spike as a transient elevation in IOP at 3 months. This was consistent with our study. Cvintal et al’s^11^ study defined a hypertensive phase as rise in IOP over 6 months from the IOP measured at 1 week, and reported a hypertensive spike in 63% of the cases. We believe this definition does not reflect our intention of describing the transient spike in IOP in the initial period after implantation (1-4 months). When comparing the average IOP in Cvintal et al’s^11^ study at 1 week, 1 month, and 3 months, the 1-month IOP appears to be an average of the 2 endpoints, which indirectly supports the notion that the M4 valve does not create a hypertensive spike at the 1-month visit. However, as in Cvintal et al’s^11^ study, the IOP at our study peaked at around 6-8 months and then slowly stabilized. The hypertensive spike difference between the three studies appears to be more based on the definition of the phenomenon, rather than differing results.

Our surgical success rate of 71.5% was significantly higher than the previous series of 53% reported by Cvintal et al,^11^ despite our more stringent surgical failure criteria of IOP >18 *vs* IOP >21. While our patient demographics was similar in proportions of complex glaucoma cases (uveitic, neovascular, chronic angle closure), our patient population was predominantly Caucasian (86.3%) *vs* (56%). However, in our study series, there was no racial statistically significant predilection for surgical failure. The number of the postoperative medications at 12 months (1.2) is also equivalent between our studies.

About 13.9% of patients required surgical intervention for complications related to the implant, most commonly for tube occlusion and exposure. It was noted that the first implants shipped to surgeons had an anterior area of the plate that was raised above the globe, rather than flush, and over time, had the propensity to erode. For all subsequent shipments of the implant, the anterior plate was flush with the sclera, which eliminated the erosion problem; however, our data reflect this plate design evolution. Moreover, since the plate area of erosion was a nonessential part of the implant once the implant was set in place by fibrosis, it could easily be cut out and removed and the area covered by a patch graft and closed primarily without further risk of erosion.

In the first 3 months of the M4 implantation, the rate of immediate postoperative hypotony and iris tube obstruction were increased compared with other Ahmed tubes at our institution. This led to alteration of surgical technique where an extra step was taken at the end of the procedure where the anterior chamber was filled during surgery with Healon ophthalmic viscoelastic device (Abbott Medical Optics, Santa Ana, CA) and the eye was kept dilated with cyclopentolate 0.5% twice a day for 1 month instead of 1 week. Cyclopentolate was used to dilate the iris and posteriorly rotate the ciliary body to avoid tube obstruction, which was seen in our first few cases. There appears to be something innate to the M4 that promotes filtration leading to early hypotony compared with the other Ahmed valves. The valve mechanism of the Ahmed valve is unchanged from the previous models; however, vascular ingrowth into the porous plate may be forming a better filtration system thus, leading to a higher rate of hypotony.

## CONCLUSION

Based on our experience, the M4 valve appears to have less of a hypertensive phase compared with the other Ahmed class valves with a similar safety profile. While a success rate of 71.5% was achieved at final follow-up, failure rate steadily increased over time similar to other Ahmed valves. While our study is limited by its retrospective nature, our large patient population and f ollow-up appear to provide important insight into the novel porous design of glaucoma drainage implants. More long-term follow-up studies are needed to better assess whether the M4 design poses more long-term complications to the patients compared with the traditional valves.

## CLINICAL SIGNIFICANCE

While the M4 production has been discontinued, the porous design of the M4 may avoid a pressure spike in the Ahmed valve class and warrants future investigation for valve design.
